# Supplementary material for: Chlamydomonas reinhardtii, an Algal Model in the Nitrogen Cycle
Source: Plants (Basel). 2020 Jul 16;9(7):903. doi: 10.3390/plants9070903 (PMC7412212; doi:10.3390/plants9070903)
Supplement: Supplementary file 1 [file plants-09-00903-s001.pdf]

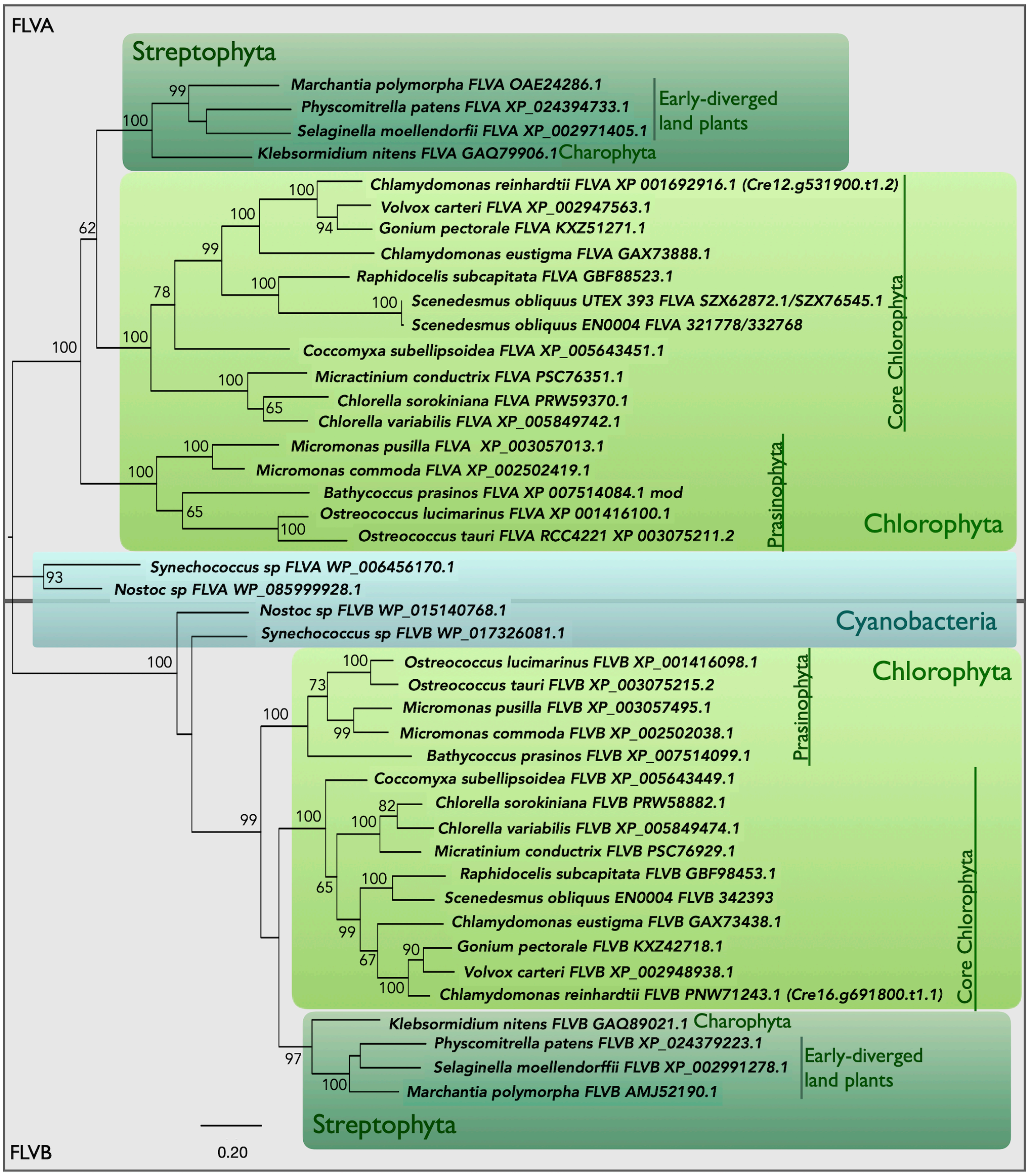

Figure S1. Evolutionary relationships of putative FLV proteins. The tree was obtained using the Maximum Likelihood method and JTT matrix-based model [1] conducted in the software MEGAX [2]. The alignment was performed using Clustal method. Numbers in branches indicate the bootstrap values.

1. Jones D.T., Taylor W.R., and Thornton J.M. The rapid generation of mutation data matrices from protein sequences. *CABIOS.* **1992**, *8*, 275-282.
2. Kumar S., Stecher G., Li M., Knyaz C., and Tamura K. MEGA X: Molecular Evolutionary Genetics Analysis across computing platforms. *Mol. Biol. Evol.* **2018,** *35*, 1547-1549.

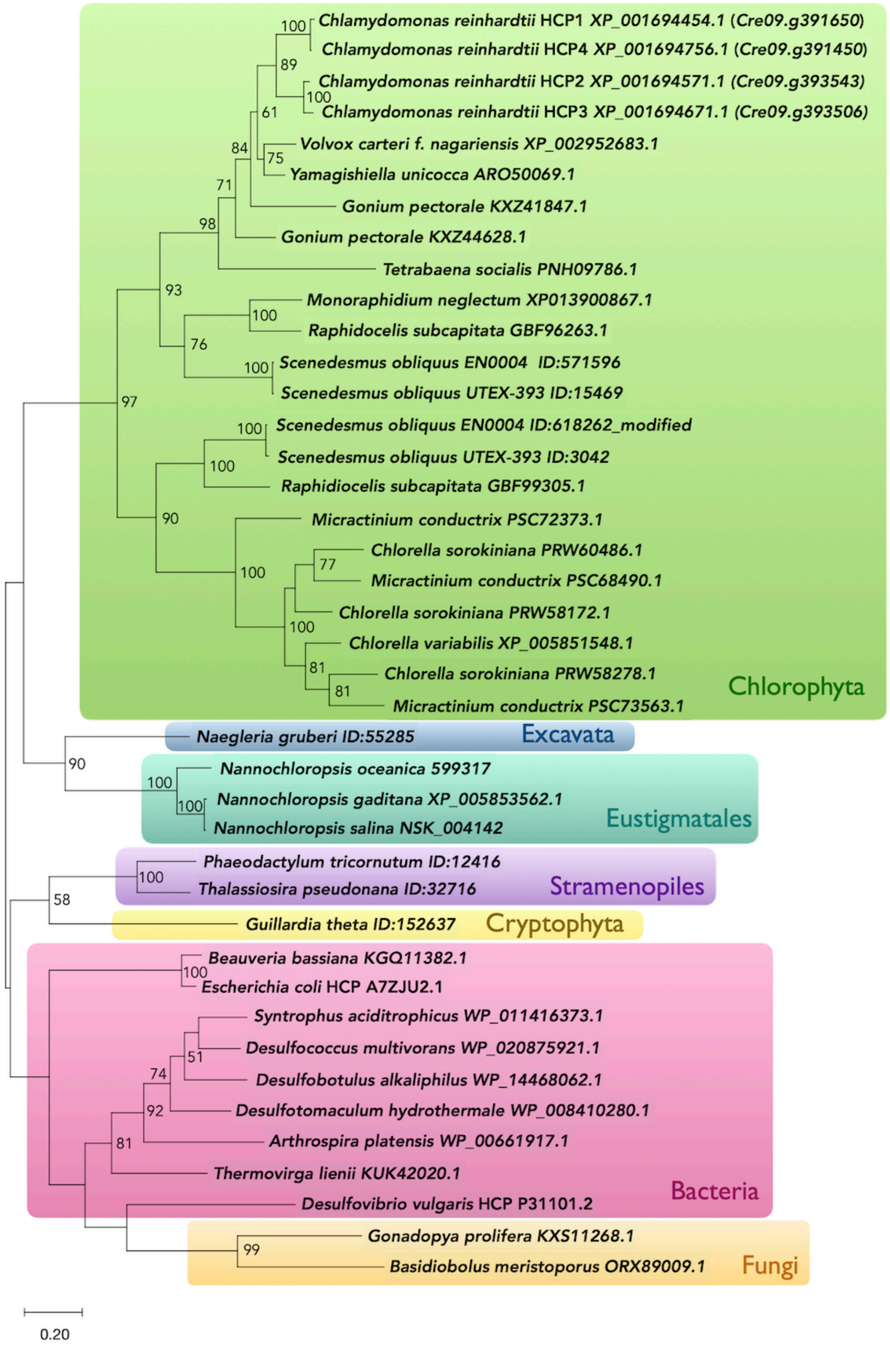

Figure S2. Evolutionary relationships of putative HCP proteins. The tree was obtained as indicated in Figure S1.

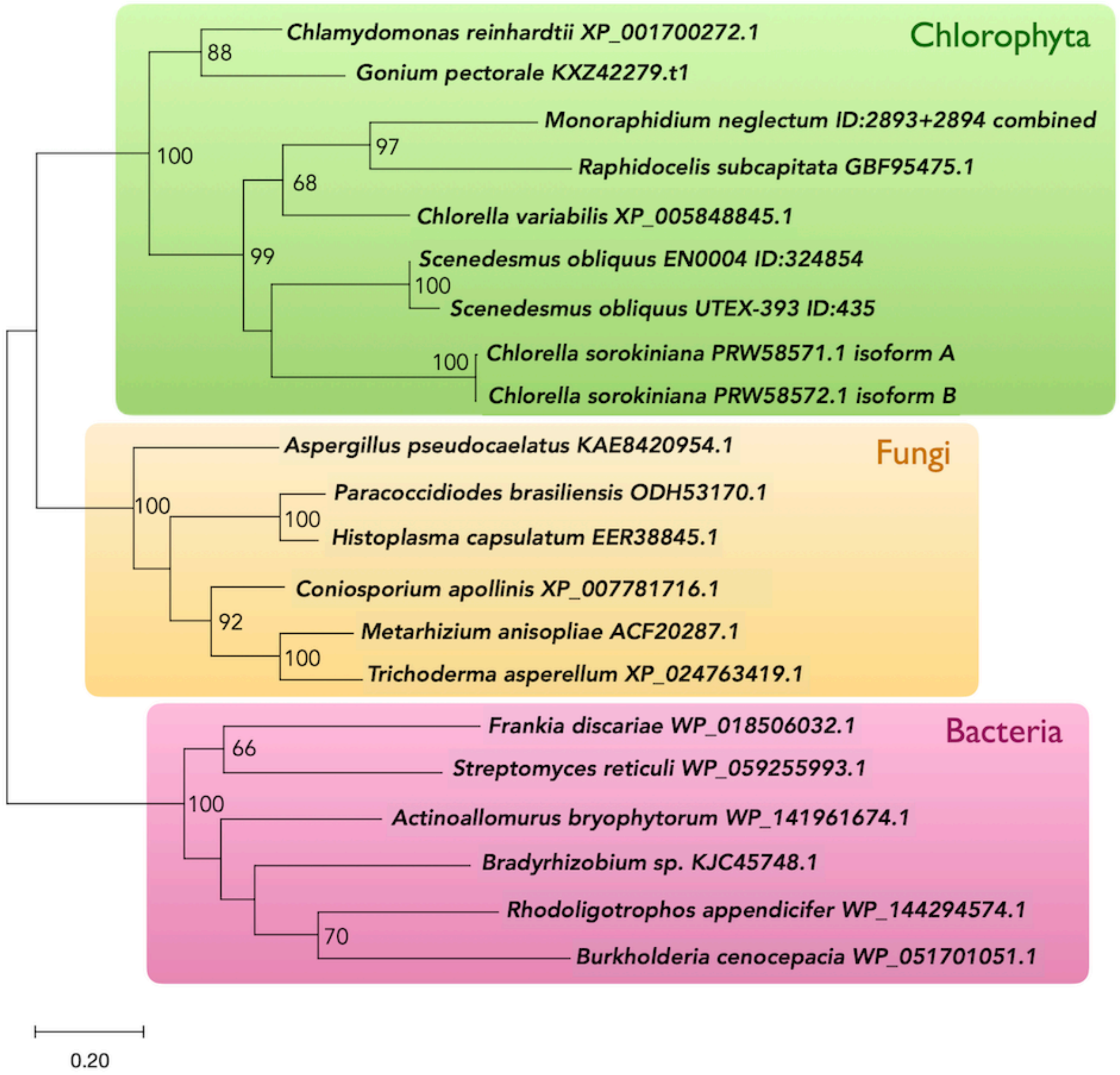

Figure S3. Evolutionary relationships of putative CYP55 proteins. The tree was obtained as indicated in Figure S1.

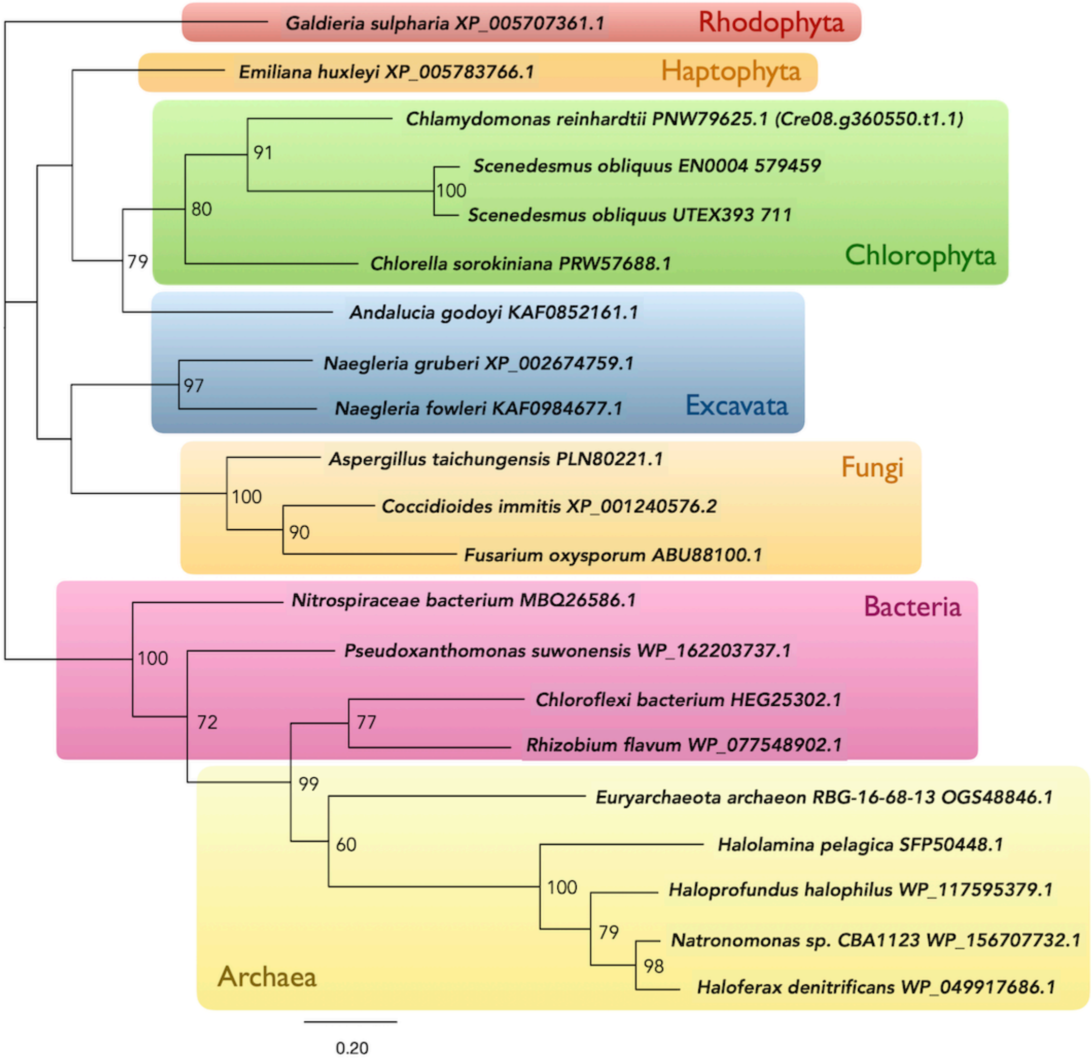

Figure S4. Evolutionary relationships of putative NIRK proteins. The tree was obtained as indicated in Figure S1.
